# Detecting the priority areas for health workforce allocation with LISA functions: an empirical analysis for China

**DOI:** 10.1186/s12913-018-3737-y

**Published:** 2018-12-12

**Authors:** Bin Zhu, Yang Fu, Jinlin Liu, Rongxin He, Ning Zhang, Ying Mao

**Affiliations:** 1 School of Public Policy and Administration, Xi’an Jiaotong University, Xi’an, 710049 China; 2 Department of Public Policy, City University of Hong Kong, Hong Kong, 999077 China; 3 College of Management, Shenzhen University, Nanhai Ave 3688, Shenzhen, Guangdong China

**Keywords:** Health workforce, Local indicators of spatial association, Spatial autocorrelation, Local Moran’s I, China

## Abstract

**Background:**

Health workforce misdistribution leads to severe inequity and low-efficiency in health services in the developing countries. Targeting at China, this research aims to reveal, visualize and compare the geographical distribution patterns of different subtypes of urban and rural health workforce and identify the priority regions for health workforce planning and allocation policies designing.

**Methods:**

The health workforce density (workforce-to-population ratio) is adopted to represent the accessibility to health workforce in each geographical unit. Besides a descriptive geography of health workforce as a whole, the local indicators of spatial association (LISA) are used to explore the spatial clusters of different subtypes of health workforce, which are visualized by geographical tools.

**Results:**

Results reveal that regional disparities and spatial clusters exist in China’s health workforce distribution, with different types of workforce exhibiting relatively different spatial distribution characteristics. Besides, huge urban-rural disparities are found in the distribution of health workforce in China. Unexpectedly but intriguingly, most of the high-high and high-low cluster area of urban health workforce are concentrated in the western China (Xinjiang, Xizang etc.), indicating the relative abundant stock of urban health workforce in these units, while the low-low and low-high cluster area of different types of urban health workforce are mainly distributed in middle China. Regarding the rural health workforce, there is an obvious and similar low-low and low-high clustering pattern in western provinces (Sichuan, Yunnan) for the licensed doctors, pharmacists, technologists, which play a critical role in health services delivery.

**Conclusions:**

Different types of health workforce displayed distinct spatial distribution patterns, while the misdistribution of rural health workforce imposed more challenges to the Chinese health sector due to its poorer stock and more disadvantaged positions of backward regions (i.e., low-low and low-high cluster area). Subtype-specific and region-oriented health workforce planning and allocation policies are suggested to be made, aiming at the urban and rural health workforce respectively, by prioritizing the identified low-low and low-high cluster areas.

**Electronic supplementary material:**

The online version of this article (10.1186/s12913-018-3737-y) contains supplementary material, which is available to authorized users.

## Background

Human well-being is highly reliable on the basic health conditions. However, the improvement of health nationwide is never an easy task. Since the 21st century, the world has been facing unprecedented health challenges ranging from demographic changes, aging populations, a much broader spectrum of infectious disease outbreaks (such as SARS (Severe Acute Respiratory Syndromes), Ebola, and influenza) to rising rates of non-communicable diseases [1]. These challenges do not only take a heavy toll on the healthcare system but also threaten the economic and social development. How we respond to these challenges profoundly influences the health of general population in one country. On the frontier against these challenges are variegated health workforce [2]. Although the financial and equipment investment play an important role in improving population health, health workforce is the core in a country’s health system as all the successful health interventions cannot be achieved without skillful health workers [3–6]. Many studies have proven the significant relationship between health workforce density (HWD, workforce-to-population ratio) and health outcomes [6, 7]. It is also widely recognized that health human resources are central to achieving health reform goals and universal health coverage [8, 9].

As the third-biggest country in the world, the problem of the health workforce in China was deemed as a “crisis” due to the backward health system and medical education during the past decades [4, 10]. In order to change the situation, the government of China implemented a new round of large-scale health reforms (also referred to as New Medical Reform) in 2009, which gained enormous political and financial support [11]. After years of efforts, the quantity of health workforce in China increased remarkably. According to the China Health and Family Planning Statistical Yearbook 2017 [12], the health workforce stock in China has reached 11.2 million in 2016, with a growth of approximately 0.5 million per year since the New Medical Reform. However, the scope of institutional efforts to improve health service quality must go beyond the growth in the number of health workforce because the problem of health workforce misdistribution has become increasingly intricate due to urbanization. In recent decades, China’s blossoming economy has compelled millions of Chinese villagers to move into cities for better living. Accordingly, more and more health resources have been poured into densely populated cities at the expense of those who live in rural and remote areas. As inappropriate allocation of resources contributes greatly to inequities in healthcare services [13], health workforce misdistribution has become one of the challenges faced by the Chinese health sector.

Up to now, the academic world has provided much evidence to explain the health workforce misdistribution in China. For example, Liu et al. [14] used the number of health technicians to measure the levels of the equity in health workforce distribution in China and concluded that the overall equity of health workforce distribution improved gradually after 2009. Chen et al. [15] analyzed the distribution of community health workforce based on the survey data from 190 health service centers in China and found the inequity in quality and geographic distribution. Zhou et al. [16] computed the inequalities in health workforce distribution in different stages of health reforms and found that the overall inequalities in the distribution of health workers decreased to the lowest level in 2000, and then increased gently until 2011. Jin et al. [17] used Gini coefficient to measure the equity in the distribution of medical personnel in 2013 and concluded that China’s distribution of health workforce is demographically, rather than geographically, more equal. Song et al. [18] explored the inequity in the distribution of paediatricians and paediatric nurses with Lorenz curve, Gini coefficient and Theil index. More similar studies can be found in the provincial inequity analysis of health technicians distribution [19] and urban-rural inequality analysis of the distribution of doctors and nurses [3].

Even though these studies help us understand the health workforce distribution in China from a global perspective, they still bear some limitations. First, existing research methods, such as the Gini coefficient and Theil index, both face shortcomings. For example, to measure the degree of equity in health workforce distribution at the province level, the first step is often to sort provincial units on the basis of HWD. However, space position information is lost in the process of sorting. As a result, space position information has been overlook by the existing methods, resulting in difficulties in identifying the priority areas for health workforce allocation. Second, existing studies mostly investigate the distribution of a single subtype of health workforce without a comparison of different kinds of health workers. Up to now, only the distribution of several limited types of health workforce has been studied. Moreover, few compare their distribution patterns and explore the similarity and difference, which limits our understanding of the health workforce distribution.

To fill the research void and have a solid knowledge of the geography and distribution patterns of health workforce, this study aims to introduce the Local Moran’s I, which is one of the Local Indicators of Spatial Association (LISA), to describe, visualize and compare the spatial distribution of urban and rural health workforce in China. We hope that findings of this study can provide a basis for planning more effective regional-oriented distribution policies and promote a more equitable distribution of health workforce. The following parts are arranged as follows: Section 2 introduces the classification of health workforce in China. Section 3 gives a detailed introduction about the data and methods. Section 4 displays the results with tables and maps. Section 5 discusses the priority areas for health workforce allocation and relevant policy application prospect. Section 6 draws the conclusions.

## Health workforce classifications in China

The Chinese health sector developed its own classification system of health workforce, which is different from the internationally commonly used ISCO-88 (the third version of the International Standard Classification of Occupations) and ISIC (International Standard Industrial Classification) [6]. There are 5 composing sections in the health workforce in China, namely, health technicians, logistics technical workers (LTW), administrative personnel (AP), and other technical personnel (OTP), village doctors & assistants (VDA), among which health technicians can be further divided into licensed doctors (LD), registered nurses (RN), pharmacists, technologists, and other medical technical personnel (OMTP), which are direct provider of health services. The detailed definitions of these 9 categories of health workforce are listed in the following Table 1. In addition, the urban health workforce is classified as those professional who are working at the municipal districts and rural health workforce refers to the rest. It is worth noting that this study only focuses on 8 subtypes of health workforce (except VDA) which are working in both urban and rural areas.

**Table 1:** Health workforce classification and definition in China

Category		Definition
Health technicians	Licensed doctors (LD)	Licensed Doctors include doctors with the certificate of medical practitioner and the certificate of assistant medical practitioner who are engaged in medical practice, exclusive of those in the managerial positions.
	Registered nurses (RN)	Registered Nurses are the nurses with a registered nurse license who are engaged in nursing practice, exclusive of the nurses in managerial positions.
	Pharmacist	Pharmacists include all levels of pharmacists from chief pharmacist to assistant pharmacist.
	Technologist	Technologists include medical laboratory technicians and diagnostic imaging technicians of different levels (including chief technologist, associate chief technologist, attending technologist etc.).
	Other medical technical personnel (OMTP)	OMTP includes various interns for medical practice (doctor interns, nurse interns, technologist interns etc.).
Other technical personnel (OTP)		Professionals responsible for technical, research and health education & promotion support.
Administrative personnel (AP)		Professionals responsible for the management duties and daily operation of the hospitals and clinics, including personnel engaged in health care, disease control, medical supervision, medical research and medical education duties Other personnel in party, government, human resources, finance, IT and security affairs.
Logistics technical workers (LTW)		Professionals responsible for repairing and logistic services, including technicians and ordinary workers.
Village doctors & assistants (VDA)		Those doctors who serve in the village clinics with the “village doctor” certification are listed as the village doctors while those serving at the same places without the certification are listed as assistants.

## Data and methods

### Data sources and indicators

The provincial year-end data of health workforce in 2016 were obtained from the China Health and Family Planning Statistical Yearbook 2017, with Hong Kong, Macau and Taiwan being excluded due to data accessibility and inconsistencies of statistical caliber. Please see the Additional file 1: Figure S1 for the Chinese administrative divisions and their names. The urban and rural health workforce and population data in each provincial unit in 2016 can be found in Additional file 2: Table S1 and Additional file 3: Table S2, respectively. The density data of different types of urban and rural health workforce in 2016 can be found in Additional file 4: Table S3.

In this study, we adopt HWD (formula 1) for representing the health workforce accessibility in urban and rural areas. It is calculated as the workforce-to-population ratio, represented by the number of health workforce per 1000 population in WHO reports, national and international archives & documents and academic writings [4, 20, 21].

1 $$ \mathrm{HWD}=\frac{\mathrm{the}\ \mathrm{number}\ \mathrm{of}\ \mathrm{health}\ \mathrm{workforce}\ \left(\mathrm{Urban}/\mathrm{Rural}\right)}{\mathrm{the}\ \mathrm{number}\ \mathrm{of}\ \mathrm{population}\ \left(\mathrm{Urban}/\mathrm{Rural}\right)} $$

### Local indicators of spatial association (LISA)

Anselin [22] distinguished the spatial attributes of data into two types: spatial heterogeneity and spatial autocorrelation. Spatial heterogeneity refers to the inconsistent relationship between dependent variable and independent variable (i.e., geographically varying coefficients between dependent variable and independent variable). Spatial autocorrelation means the pairwise correlation between georeferenced observations for a specific indicator [23]. Simply put, the indicator in one unit is influenced by the same indicator in other units. This study only involves the spatial autocorrelation as we focus on one specific indicator—HWD. If exists, spatial autocorrelation of HWD means the clustering or concentration tendency of health workforce [24].

The spatial autocorrelation is usually explored by the global and local indicators of spatial association. The global indicators of spatial association (GISA) give a direct reflection on the unevenness of the geographical distribution of observations as a whole. However, the GISA only provides limited understanding of the health workforce distribution as the health workforce clusters differ across localities and regions. The LISA separate the global ones into sub indicators of each location, with its sum for all the units being proportional to the corresponding global indicator. The main functions of LISA lie in the detection of local spatial clusters, which are widely used in health research [25–27]. The process of cluster detection with LISA functions can be generally divided into three steps.

#### Step 1: Constitution of the spatial weight matrix

Before the spatial autocorrelation analysis of the target geographical units, a spatial weight matrix has to be established, which illustrates the location information of the geographical units. This study adopts a widely used strategy to construct the spatial weight [24], a binary contiguity matrix with the rook criterion, i.e., spatial neighboring criterion based on border sharing. For example, in the following formula (2), i and j are provincial units in China. If they are adjacent provincial units, the value of *W*_*ij*_ will be 1 while if these two units share no border, the value of *W*_*ij*_ will be 0. We have row-standardized the spatial weight matrix to control the influences from the number of bordering units.

2 $$ {W}_{ij}=\left\{\begin{array}{c}1\ \mathrm{if}\ \mathrm{two}\ \mathrm{geographical}\ \mathrm{units}\ i\ \mathrm{and}\ j\ \mathrm{share}\ \mathrm{borders}\\ {}0\ \mathrm{otherwise}\kern17.5em \end{array}\right. $$

#### Step 2: Identification of the local spatial clusters

Local spatial clusters are identified as those areas for which the LISA is significant [28]. There are many pairs of global-local indicators illustrating the uneven geographical distribution of targeted indicators and Moran’s I (global and local) is one of the most widely used in health-related studies, especially the clustering of diseases cases [29–33]. It owns the advantage to display various types of spatial distribution characteristics [34]. In this case, we need to investigate the local Moran’s I of the geographical units to draw a clear picture of the variegated scenarios of different local spatial distribution characteristics. It can be explained by the following formula (3):

3 $$ {\mathrm{Local}\ \mathrm{Moran}}^{\prime}\mathrm{s}\ \mathrm{I}=\frac{\left({x}_i-\overline{x}\right)}{{\mathrm{m}}_0}\sum \limits_j{W}_{ij}\left({x}_j-\overline{x}\right)\kern1.25em {\mathrm{m}}_0=\sum \limits_i{\left({x}_i-\overline{x}\right)}^2/\mathrm{n} $$

In this formula, n is the number of geographical units, *x*_*i*_ is the HWD of 8 subtypes in the corresponding provincial units, while $\overline{x}$ is the average value of each subtype health workforce in China. As has been deliberated on in the previous paragraph, *W*_*ij*_ is the spatial weight matrix. It should be noted that “the operation of summing j is limited to the surrounding areas of i” [24] since the focus of the local Moran’s I only pivots to each single unit and the bordering units. m_0_ is a constant based on the estimation of the variance when applied to each geographical unit. The value of local Moran’s I varies from − 1 to 1. A positive value approaching 1 means a stronger geographical concentration of units with similar values (high-high, low-low; high values and low values are classified based on the mean value) while that approaching − 1 stands for the opposite situation (low-high, high-low clustering patterns). If local Moran’s *I* = 0, it means the random distribution of the units.

**Fig. 1:**
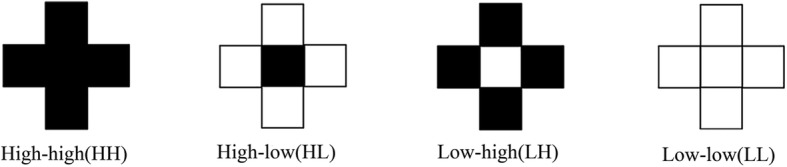
Four types of spatial clusters detected by the Local Moran’s I

However, it is important to note that the cluster in geography is different with our common understanding. The geographical clusters refer to not only the concentration of high-value units but also the low-value units. In this case, the units with relatively sufficient and inefficient stock of health workforce can both be referred to geographical clusters. The spatial clusters detected by the local Moran’s I can be divided into four types: high-high (high-density units surrounded by high-density units), high-low (high-density units surrounded by low-density units), low-high (low-density units surrounded by high-density units), low-low (low-density units surrounded by low-density units). Figure 1 is an illustration of the four types of clusters. For those high-value units with significant local Moran’s I, their cluster types are high-high (if the local Moran’s I is positive) or high-low (if the local Moran’s I is negative). For those low-value units with significant local Moran’s I, their cluster types are low-low (if the local Moran’s I is positive) or low-high (if the local Moran’s I is negative). The high type of cluster (HH and HL) indicates the abundant stock of health workforce in the central units, while the low type of cluster (LL and LH) mean exact the opposite.

#### Step 3: Summarization and visualization of the local spatial clusters

This study will use maps to reveal the differences in the density of 8 subtypes of health workforce and their spatial clustering patterns across the provincial units in China.

First, the indicators of each subtype health workforce are made into hierarchy maps with natural breaks respectively (Figs. 3 and 5). It is a widely adopted grouping method in statistics [35–37], which classifies the observations by the maximum statistical difference between groups and minimum differences within each group [38]. All the hierarchical maps of the provincial units are divided into four scales. The darker the color, the more serious the personnel insufficiency.

Second, the local Moran’s I of the indicator of each subtype are used to draw the corresponding univariate LISA cluster maps (Figs. 4 and 6), illustrating the four clustering types as mentioned in the previous paragraph. The provincial units that reach the significant level are visualized on the LISA maps into four clustering patterns.

In the end, to better understand the various and complex spatial distribution patterns of health workforce, the four clustering patterns are summed up in two groups based on the value of central position in clusters, high type of clusters (HL and HH, the central position with high value) and low type of clusters (LL and LH, the central position with low value). The occurrence of each provincial unit in high or low type of clusters across 9 subtype health workforce categories are summed up in maps (Additional file 5: Figure S2 and Additional file 6: Figure S3). They highlight the priority areas for overall health workforce planning in China.

### Software tools

GeoDa (Version 1.8.16, the University of Chicago, Chicago, IL, USA) is employed to constitute the spatial weight matrix and compute the local Moran’s I. Then ArcGIS 10.0 (Version 10.0, ESRI Inc., Redlands, CA, USA) is used to visualize the data from GeoDa. Finally, the total occurrence of provincial units in the low and high type of clusters are illustrated in the frequency maps with ArcGIS 10.0.

## Results

### Size, composition and distribution

Table 2 is a descriptive summary of the 8 categories of health workforce and their rural/urban composition. The total number of employees is 10,172,621 and its distribution in the urban and rural areas is almost even, each accounting for 53.94 and 46.06% of the whole. Licensed doctors and registered nurses are the most substantial composing parts of the health workforce, which take 31.37 and 34.48% of the health human resources in China. For the 31.37% licensed doctors, 16.20% are in urban areas while 15.17% are in the rural areas. Registered nurses concentrated more in the urban areas as well, with urban registered nurses take 20.28% of the total health workforce and their rural counterparts take only 14.20%.

**Table 2:** Summary Statistics of each type of health workforce in 2016

Category	Number			Percentage		
	Total	Urban	Rural	Total	Urban	Rural
Licensed doctors	3,191,005	1,647,676	1,543,329	31.37%	16.20%	15.17%
Registered nurses	3,507,166	2,063,019	1,444,147	34.48%	20.28%	14.20%
Pharmacist	439,246	228,161	211,085	4.32%	2.24%	2.08%
Technologist	453,185	241,749	211,436	4.45%	2.38%	2.08%
Other medical technical personnel	863,801	347,103	516,698	8.49%	3.41%	5.08%
Other technical personnel	426,171	234,224	191,947	4.19%	2.30%	1.89%
Administrative personnel	483,198	287,296	195,902	4.75%	2.82%	1.93%
Logistics technical workers	808,849	438,089	370,760	7.95%	4.31%	3.64%
Total	10,172,621	5,487,317	4,685,304	100.0%	53.94%	46.06%

**Fig. 2:**
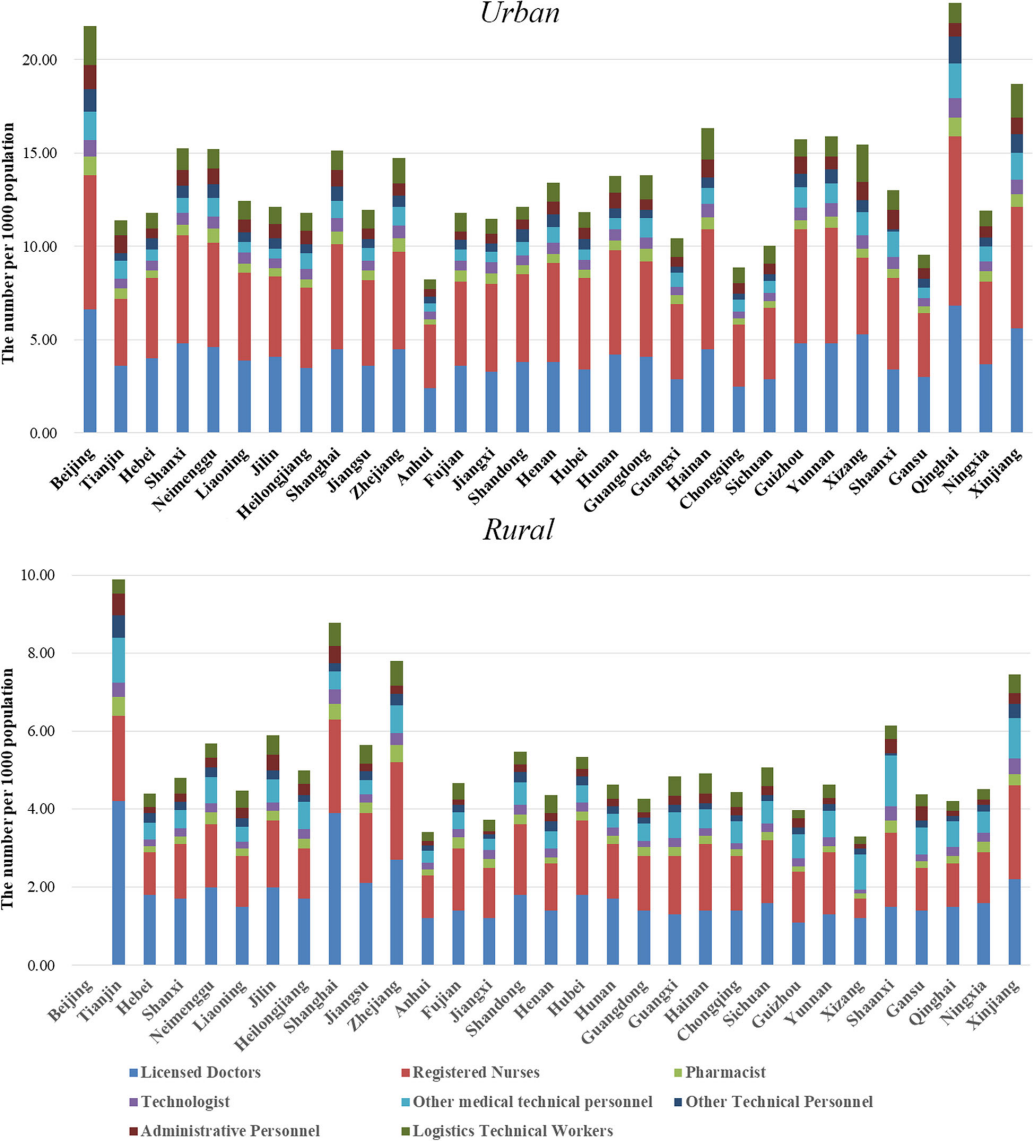
Size, composition and distribution of urban and rural health workforce in China in 2016. All the health workforce in Beijing was classified as urban health workforce in 2016

We further divide the health workforce allocation in the urban and rural areas, which is shown in Fig. 2. The division of 8 categories of health human resources in the urban and rural regions is more revealing since it the urban-rural divide is discrepant in China’s health resources. Beijing is an exception as all health workforce in Beijing is categorized as urban employees. Other than Beijing, Qinghai has the highest density of licensed doctors, which is followed by Xinjiang and Xizang. However, the urban-rural divide in terms of licensed doctors in these provincial units is stunning, with the rural density in these units being relatively low. As to registered nurses, the highest density in urban areas is detected in the provincial units of Qinghai, Xinjiang and Yunnan whereas the density in rural areas is extremely low in Anhui, Chongqing and Gansu. The rural-urban gap is even more dramatic regarding the distribution of registered nurses within and across the provincial units. Other categories only take a minor proportion of the entire health workforce yet similar discrepancies are found in between the urban and rural areas.

### Detection of local spatial clusters for urban areas

**Fig. 3:**
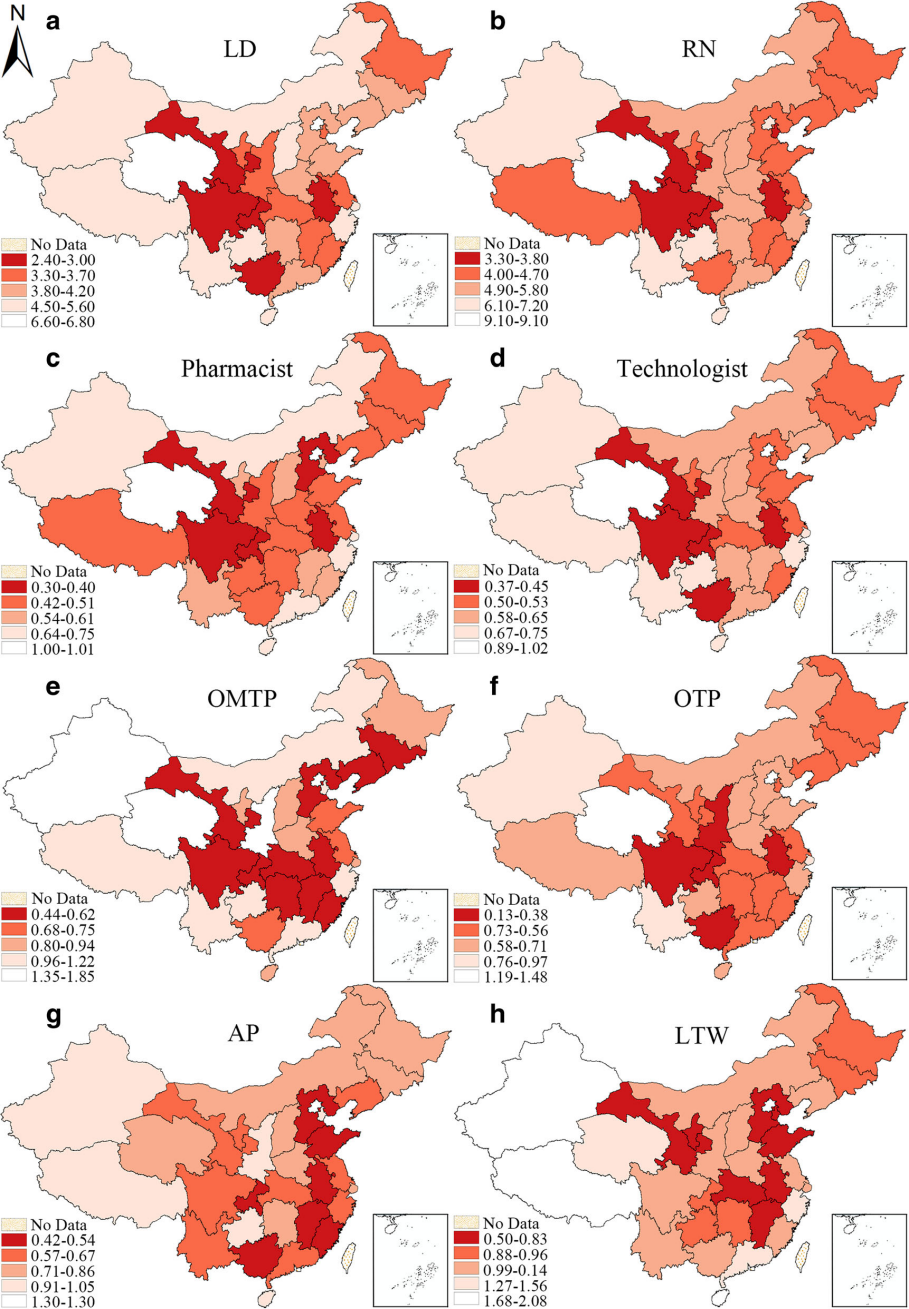
Hierarchy map of the HWD of 8 subtypes of health workforce in urban areas in 2016. (**a**-**h** represent different subtypes of health manpower, which have been specified on the maps, the same above)

Figure 3 is the hierarchy maps of HWD of all 8 subtypes of health workforce in urban areas, the maps demonstrate the density of urban health workforce in all the provincial units with the natural break that split all units into four scales. Generally, there was an obvious lack of urban health workforce in some provinces (Sichuan, Gansu, Chongqing, Anhui etc.). Some sub-categories of health workforce shared a similar distribution pattern. For instance, Beijing, Qinghai, Yunan and Guizhou had a higher density of licensed doctors, registered nurses, pharmacist and other medical professionals.

**Fig. 4:**
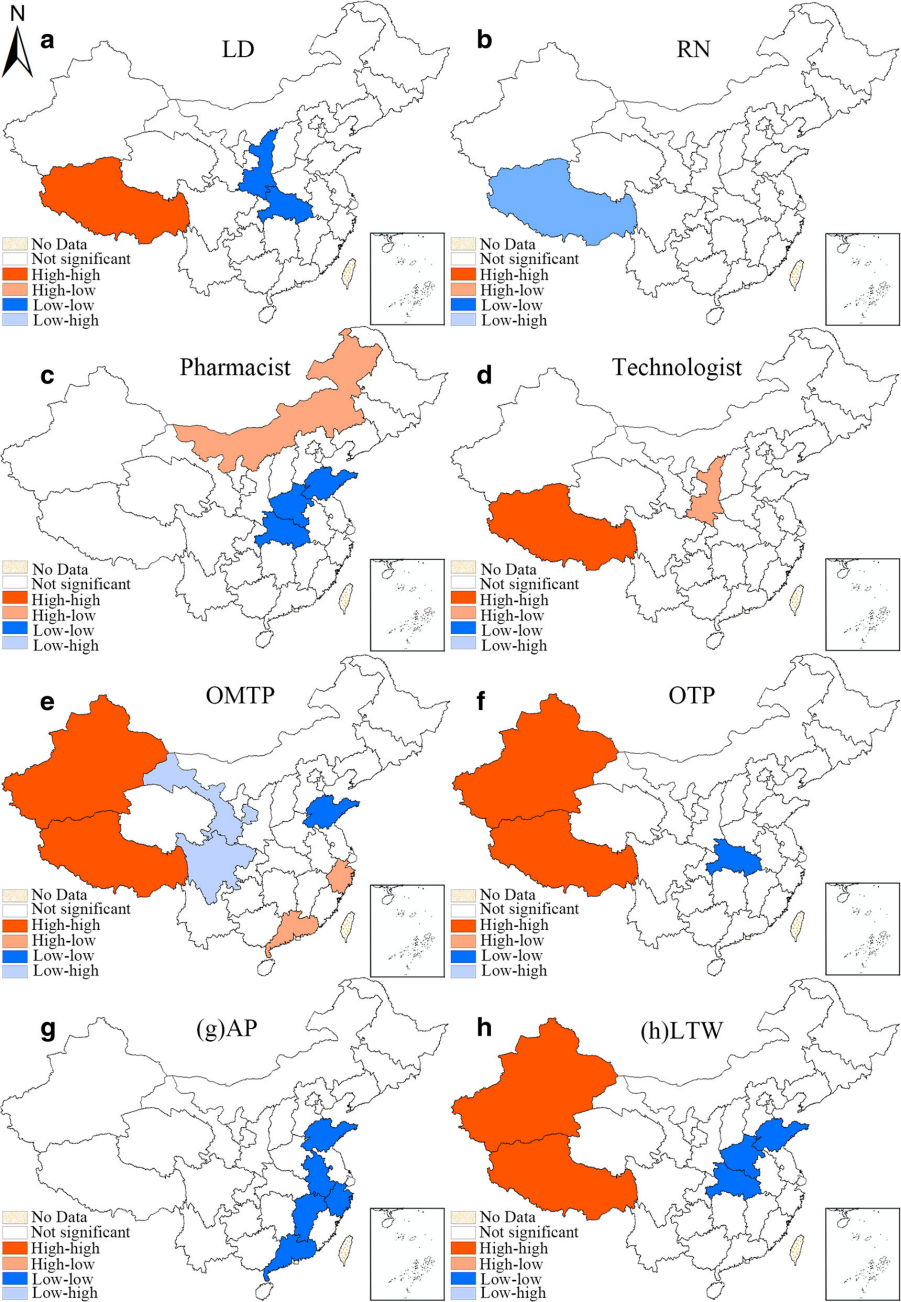
Univariate LISA cluster maps of the HWD of 8 subtypes of health workforce in urban areas in 2016. (**a**-**h** represent different subtypes of health manpower, which have been specified on the maps, the same above)

Figure 4 is the univariate LISA maps of all 8 subtypes of urban health workforce. The spatial clustering patterns of different types of urban health workforce were revealed in these maps. There was a HH cluster of licensed doctors in Xizang and a LL cluster in Shaanxi and Hubei. As to registered nurses, only a LH cluster was detected in Xizang. The LL cluster of pharmacist was significant in Hubei, Henan and Shandong while the HL cluster was significant in Neimenggu. There was a HH (Xizang) cluster and HL cluster (Shanxi) in the distribution of technologist. The HH clustering patterns were similar for OMTP, OTP and LTW, with Xizang and Xinjiang identified as the HH cluster units whereas for AP, there was only LL cluster found, which were Guangdong, Jiangxi, Anhui, Zhejiang and Shandong. The maps that summarize the frequency of cluster occurrence among the 9 subtypes of urban health workforce can be found in Additional file 5: Figure S2.

### Detection of local spatial clusters for rural areas

**Fig. 5:**
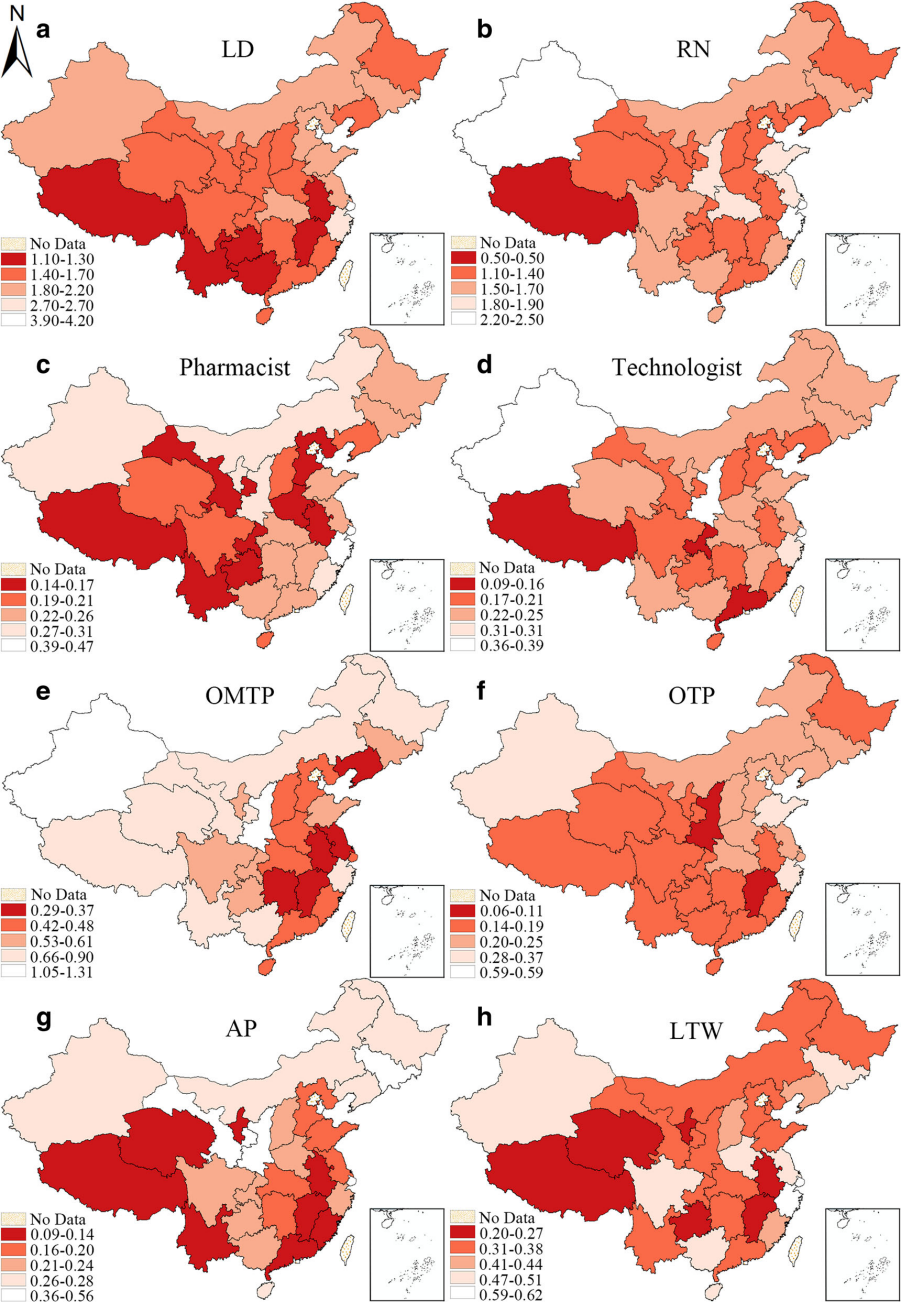
Hierarchy maps of the HWD of 8 subtypes of rural health workforce in 2016. (**a**-**h** represent different subtypes of health manpower, which have been specified on the maps, the same above)

Figure 5 is the hierarchy maps of HWD of all 8 categories of rural health workforce. The spatial distribution patterns of the subtypes were very similar. As to licensed doctors, for instance, the provincial units with higher HWD were Tianjin, Shanghai and Shandong in the eastern region, while the low-HWD provinces were concentrated in the southwestern regions. The high-HWD and low-HWD provincial units of registered nurses were different only in some provinces but still shared some similarities with that of licensed doctors. It was the highest in Tianjin, Shanghai and Zhejiang and the lowest in Xizang, Guangdong and Chongqing. This distribution pattern could also be found among other subtypes. Key western provinces like Xinjiang, Shaanxi and Gansu revealed great scarcity in terms of all types of rural health workforce.

**Fig. 6:**
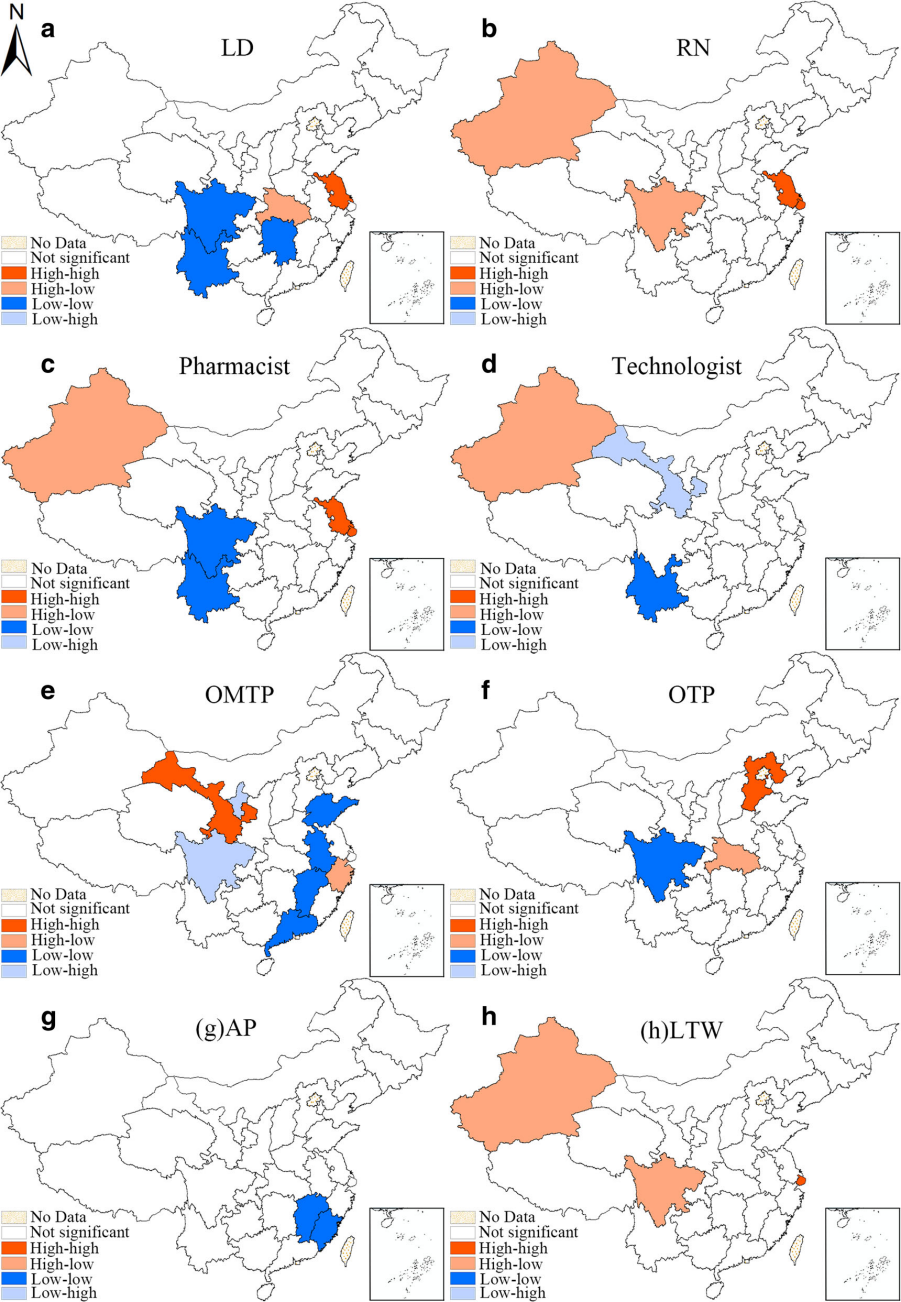
Univariate LISA cluster maps of the HWD of 8 subtypes of rural health workforce in 2016. (**a**-**h** represent different subtypes of health manpower, which have been specified on the maps, the same above)

Figure 6 is the univariate LISA maps of all 8 subtypes of rural health workforce. Regarding licensed doctors, there was an obvious LL clustering pattern in southwestern provinces (Sichuan, Yunnan and Hunan), while a HH clustering pattern was found in eastern China (Zhejiang). As to registered nurses, HH patterns were found in Jiangsu and Shanghai while LH clusters were detected in Sichuan and Xinjiang. The spatial autocorrelation of pharmacists was similar with that of licensed doctors but has some minor mutations, with the LH cluster areas found in Xinjiang. The pattern was still similar with regard to technologist. For the other five minor groups, the spatial autocorrelation patterns varied but shared lots of similarities with other types of rural health workforce. The maps that summarize the frequency of cluster occurrence among the 8 subtypes of rural health workforce can be found in Additional file 6: Figure S3.

## Discussion

Nowadays, recent developments in geography and related software enables more and more health scholars to associate geographic location information with health-related data [39]. By means of the LISA functions, this study explores, visualizes, and compares the spatial distribution patterns of 8 subtypes of urban and rural health workforce in China, which is beneficial for evidence-based policy-making for health workforce allocation to promote a balanced and equitable distribution of health workforce.

As reported previously, various types of spatial clustering can be observed in those univariate LISA cluster maps. The appearance of all four types of spatial clusters reveals the complex distribution characteristics of health workforce in China. Among all the four types of spatial clusters (HH, LH, HL, and LL), the areas in the low type (LL and LH) of clusters demand more attention as their appearance indicates the lack of health workforce in central and/or surrounding units. Of course, more attention should be paid on licensed doctors, registered nurses, pharmacists and technologists are much more important due to their direct, significant role in healthcare delivery. For instance, the density of urban licensed doctors in Shaanxi and Hubei showed LL cluster feature, indicating the lack of urban licensed doctors in them and their surrounding areas. While when it comes to the urban registered pharmacists, Shandong, Henan and Hubei stand out in the univariate LISA maps and displayed LL cluster feature. These provincial units should exactly be the priority areas of health workforce planning.

Unexpectedly but intriguingly, the density of urban health workforce in some geographical remote provinces (Xinjiang, Xizang etc.) in the western China displayed high-high cluster feature, indicting the abundant stock of urban health workforce in these units. While when it comes to the rural health workforce, some other western provincial units, like Yunnan and Sichuan, are always displaying low-low cluster feature for subtypes of rural health workforce. Besides, Xinjiang exhibited a high-low type of spatial clustering pertaining to the distribution of registered nurses, pharmacists and technologists, indicating the critical lack of these types of rural health workforce in its neighboring units (Xizang, Qinghai and Gansu). As quantity does not equate quality, these disadvantaged areas may have not only a lower density of rural health workforce but also less well-educated workforces [8]. The striking differences between urban and rural health workforce densities in these western units remind us to rethink the effectiveness and rationality of previous health workforce allocation policies. All the time, these geographical remote provinces have been deemed to be suffering disadvantages of attracting and retaining healthcare workers, and policy preferences are often given to those areas to improve the health services accessibility in these units. However, as it turns out, most of the health workforce in these units is concentrated in urban areas, resulting in more huge urban-rural differences. In other words, even though much policy preferences have been given to some western units, while these policies owned limited effects in allocating health workforce to the rural areas. In addition, the vast area further reduces the accessibility to health workforce in rural west China. For example, the four largest provincial units in China account for about 50% of the total area but only 4% of the population [40], resulting in the lower accessibility to health services under equivalent health workforce coverage level.

We can draw two take-away lessons from abovementioned spatial clusters. First, Based on the results, different types of health workforce displayed various types of spatial clustering patterns, which may be attributable to the different supply channel and training requirements of different subtypes of health workforce. This calls on subtype-specific health workforce planning and allocation policies are essential to balance the health workforce distribution. Second, China faces a tougher challenge to equally distribute rural health workforce than urban health workforce among provinces. In spite of the huge urban-rural gaps in the densities of urban and rural health workforce, more administrative units in the western China are identified as low-low cluster area in the distribution of rural health workforce. They are disadvantaged in various aspects (e.g., the education, attraction, and retention of health workforce), making them more difficult move out the low-low cluster area.

Such lessons provides a more solid and specific basis for making corresponding health workforce planning and allocation policies towards those LL and LH cluster areas. This can commonly be achieved by expanding the government and social health expenditure and strengthening the medical education system through medium-term and long-term plans [2]. Besides, the detected LL and LH clusters are exactly the objective areas for existing area-targeted health workforce programs in China. For instance, the “Rural-oriented Medical Education Scheme (RMES),” an ongoing education program which only recruits medical students with a rural background to channel trained medical personnel into the rural and remote areas. RMES provides scholarships and tuition waiver in return for obligated medical service in the rural township hospitals of western and middle regions in China. It has been proven to be an effective approach since it’s far more difficult to urge the health workers in other provinces and urban areas to migrate into the remote provinces and the countryside [41, 42]. The research findings will sharpen the focus of RMES by illuminating the most in need subtype personnel in the most in need provincial units. Another example is the “Medical Pairing-assistance Scheme”, a program to pair up provincial units with and without affluent medical human resources. The research findings will make such paring more accurate by paring up the geographical regions who are most in need and who are comparatively most sufficient in certain subtypes of health workforce. For the remote or border regions which cannot cultivate or attract high-quality health workers, some expediencies should be arranged with the assistance of telemedicine. Although less effective for serious diseases, telemedicine will save the time and energy for both the patients and physicians in the subsequent visits and recover period [43]. For example, Gansu, a backward western province, built the first telemedicine consultation center in Northwest China. At present, the center covers all of the municipal hospitals and the county hospitals as well as the township hospitals in Gansu equipped with the telemedicine network, with the number of telemedicine consultations reaching 4000 cases per year [44].

As an exploratory study attempting to investigate the issue of health workforce misdistribution in China, the present study surely has limitations that should be acknowledged. Akin to previous literature, our study on health workforce is centered on its quantity without a due consideration of its quality. As quantity does not equate quality, disadvantaged areas may have not only a lower density of health workforce but also less well-educated workforces. In addition, due to data availability, this study only targets at the health workforce distribution at the provincial level, more studies at the city or county level are encouraged in the future.

## Conclusions

Evidently, China is faced with the challenge of health workforce misdistribution, which demands more attention from policy makers. Notwithstanding the heterogeneity in different kinds of health workforce, the vista that emerges is that spatial cluster is an inherent feature in the spatial distribution of health workers and it poses great challenges in the quality of health service [45]. The cluster of each type of urban and rural health workforce, its frequency, similarities, and differences between the different types would provide much more solid evidence for area-targeted and subtype-specific health policy making in China.

To achieve the balanced distribution of health workforce in China, it is important to formulate health workforce planning and implement area-targeted health workforce programs targeting for the most prioritized areas (i.e., low-low and low-high cluster areas). More importantly, the attraction and retention of rural health workforce in remote areas should never be taken lightly, especially towards the scarce subtypes. While health workforce allocation requires not only the efforts of the health sector, but also the long-term support from other sectors (education, finance, etc.) due to its long training circle.

## Additional files

Additional file 1: **Figure S1.** The Chinese administrative divisions and their names. (TIF 415 kb)

Additional file 2: **Table S1.** The number of different types of health workforce and population data in urban China in 2016. (XLSX 11 kb)

Additional file 3: **Table S2.** The number of different types of health workforce and population data in rural China in 2016. (XLSX 11 kb)

Additional file 4: **Table S1.** The density of different types of urban and rural health workforce in China in 2016. (XLSX 12 kb)

Additional file 5: **Figure S2.** Frequency of cluster occurrence among the 9 subtypes of urban health workforce (left for HH and HL clusters and right for LL and LH clusters). (TIF 697 kb)

Additional file 6: **Figure S3.** Frequency of cluster occurrence among the 9 subtypes of rural health workforce (left for HH and HL clusters and right for LL and LH clusters). (TIF 664 kb)

## Abbreviations

AP: Administrative personnel
GISA: Global Indicators of Spatial Association
HH: High-high
HL: High-Low
HWD: Health Workforce Density
ISCO-88: The third version of the International Standard Classification of Occupations
ISIC: International Standard Industrial Classification
LD: Licensed doctors
LH: Low-high
LISA: Local Indicators of Spatial Association
LL: Low-low
LTW: Logistics Technical Workers
OMTP: Other medical technical personnel
OTP: Other technical personnel
RMES: Rural-oriented Medical Education Scheme
RN: Registered Nurses
SARS: Severe acute respiratory syndromes
VDA: Village doctors & Assistants
WHO: World Health Organization
