# Supplementary figures and images for: Detecting the priority areas for health workforce allocation with LISA functions: an empirical analysis for China
Source: BMC Health Serv Res. 2018 Dec 12;18:957. doi: 10.1186/s12913-018-3737-y (PMC6292090; doi:10.1186/s12913-018-3737-y)

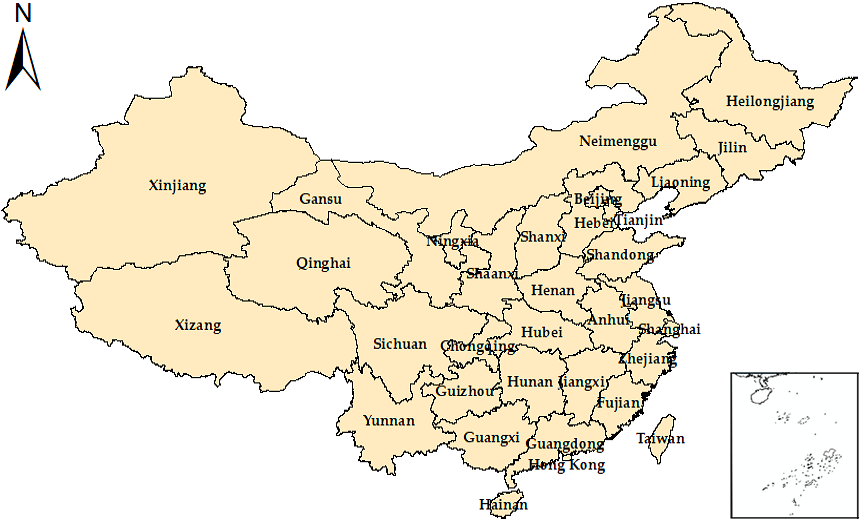

Supplement: Supplementary file 1 — Figure S1. The Chinese administrative divisions and their names. (TIF 415 kb) [file 12913_2018_3737_MOESM1_ESM.tif]

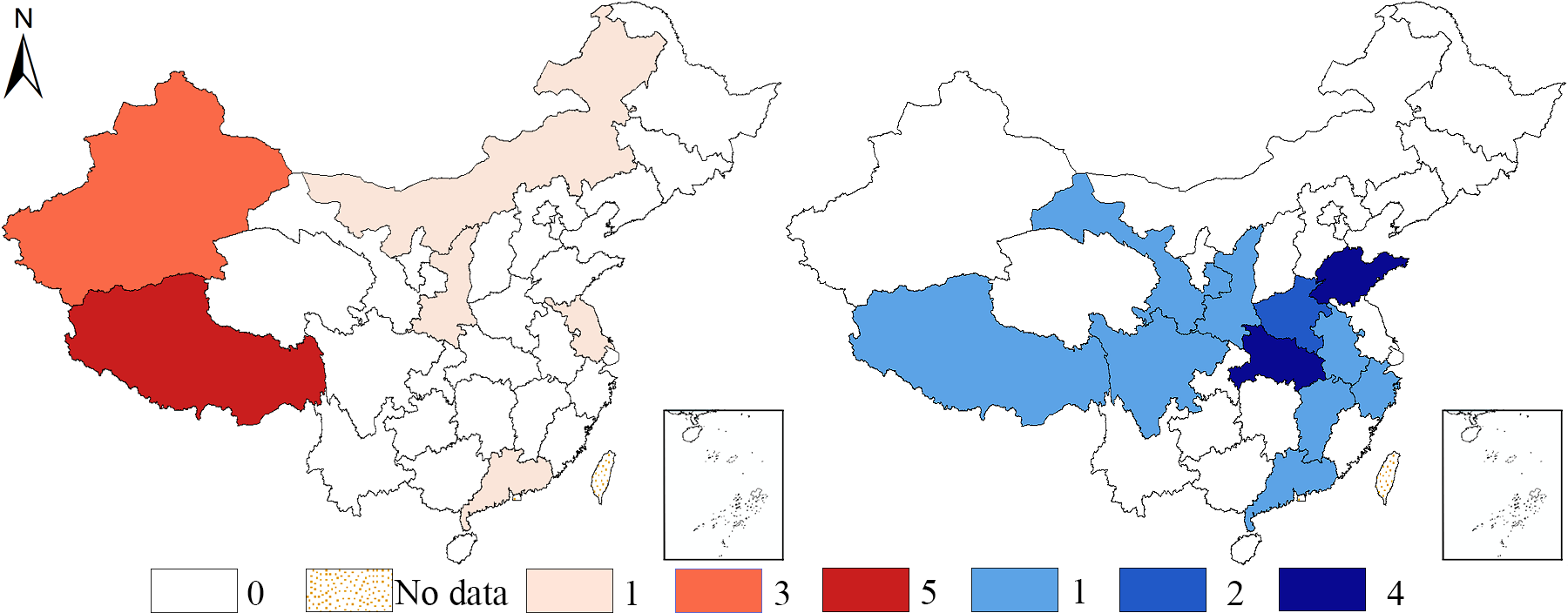

Supplement: Supplementary file 5 — Figure S2. Frequency of cluster occurrence among the 9 subtypes of urban health workforce (left for HH and HL clusters and right for LL and LH clusters). (TIF 697 kb) [file 12913_2018_3737_MOESM5_ESM.tif]

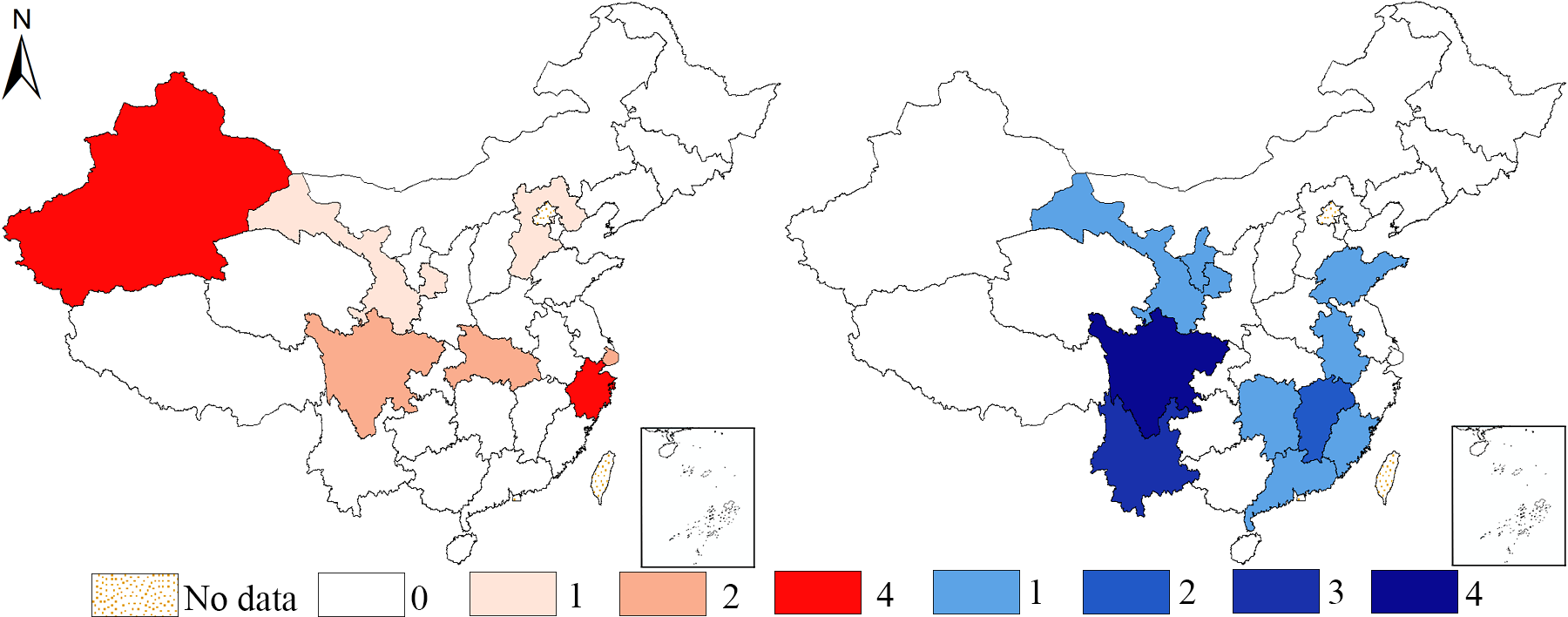

Supplement: Supplementary file 6 — Figure S3. Frequency of cluster occurrence among the 9 subtypes of rural health workforce (left for HH and HL clusters and right for LL and LH clusters). (TIF 664 kb) [file 12913_2018_3737_MOESM6_ESM.tif]
